# Biomarkers of Mercury Exposure in the Amazon

**DOI:** 10.1155/2014/867069

**Published:** 2014-04-27

**Authors:** Nathália Santos Serrão de Castro, Marcelo de Oliveira Lima

**Affiliations:** Evandro Chagas Institute (IEC), Health Surveillance Secretariat (SVS), 660990 Belém do Pará, PA, Brazil

## Abstract

Mercury exposure in the Amazon has been studied since the 1980s decade and the assessment of human mercury exposure in the Amazon is difficult given that the natural occurrence of this metal is high and the concentration of mercury in biological samples of this population exceeds the standardized value of normality established by WHO. Few studies have focused on the discovery of mercury biomarkers in the region's population. In this way, some studies have used genetics as well as immunological and cytogenetic tools in order to find a molecular biomarker for assessing the toxicological effect of mercury in the Amazonian population. Most of those studies focused attention on the relation between mercury exposure and autoimmunity and, because of that, they will be discussed in more detail. Here we introduce the general aspects involved with each biomarker that was studied in the region in order to contextualize the reader and add information about the Amazonian life style and health that may be considered for future studies. We hope that, in the future, the toxicological studies in this field use high technological tools, such as the next generation sequencing and proteomics skills, in order to comprehend basic questions regarding the metabolic route of mercury in populations that are under constant exposure, such as in the Amazon.

## 1. Introduction

Over the last 20 years, questions regarding the mercury concentrations in the Amazon have received attention from different scientific communities. During the 1990s decade, many studies reported that mercury concentration in biotic and abiotic samples from the Amazon presented higher values than from other places. Some authors judiciously reviewed those data and the source (anthropogenic or natural) of mercury in the Amazon was discussed [1, 2]. Moreover, questions about the influence of nutritional factors as potential modifiers of mercury toxicity emerged in this period [3]. As a contribution, those initial review papers presented perspectives for future studies. They emphasized the importance of biogeochemical characterization of mercury in the Amazon ecosystems, comprehension of the effect of diet on mercury toxicity, and recommended public health interventions in order to modulate the fish consumption of the Amazonian population, and, consequently, the intake of methylmercury.

Later, in the early years of the 21st century, advances in knowledge of the behavior of mercury in the Amazon could be addressed by the review papers focusing on the biogeochemical cycle of mercury in the Amazon, on epidemiological and clinical studies performed in the Tapajós basin, on the dietary intake of riverine people, and on assessment of the high amount of data produced by Brazilian researchers during expeditions along the Tocantins and Xingu basins [4–7]. Those articles unambiguously demystify some previous aspects of mercury concentration in the region. It became clear that the Amazon has its own reservoir of mercury in soil and that native people are under risk of exposure even in remote areas without any history of gold mining. This scenario creates some hypotheses to explain the apparent tolerance of mercury intoxication observed in this population. de Oliveira Santos et al. [5] postulated that intestinal polyparasitism (common among riverines) and nutritional factors might be involved in methylmercury absorption and Dorea [6] considered that fish consumption by the Amazonian population is a part of a successful strategy of health surveillance. More recently, two other studies reviewed the concentration of mercury in the biological samples of riverine populations [8, 9]. In this way, Passos and Mergler [8] argued that it is imperative to find a method for reducing exposure and toxic risk for local population, whereas Barbieri and Gardon [9] considered that the high heterogeneity between mercury concentrations in hair of the Amazonian population creates a barrier for assessing the public health implications involved with the risk of exposure. The authors propose the application of a standardized study in order to find a uniform profile of mercury exposure and determine the real human risk assessment.

The present review does not intent to describe the mercury concentration profile in Amazon ecosystem since there is a consistent literature on that issue. Our focus will be a critical analysis of recent studies about the possible molecular biomarkers for assessing mercury toxicity in the Amazonian population. Those biomarkers were contextualized in order to provide clarity regarding their function or how they are connected with other research, rather than with mercury toxicity.

## 2. GST and Mercury Exposure

The enzyme glutathione transferase superfamily (GST) has been widely studied as a potential biomarker since these enzymes have been associated with a variety of diseases, such as cancer, bipolar disorder, asthma, and diabetes [10–13]. The enzymes catalyse the conjugation of thioester bonds between the tripeptide glutathione (*γ*Glu-Cys-Gly) with electrophilic molecules. All GSTs converge into a cohesive biological pathway, mainly detoxification, but exhibit different structures and are divided into three subfamilies: microsomal, mitochondrial, and cytosolic [14–16]. The cytosolic family is the most abundant and has received much attention in toxicological studies [17, 18]. Its classification is based on substrate specificity and amino acid sequence similarity and encompasses eight classes: GSTA (alpha), GSTK (kappa), GSTM (mu), GSTP (pi), GSTS (sigma), GSTT (theta), GSTO (omega), and GSTZ (zeta) [19, 20]. This family presents a strong genetic variability among human populations and its molecular diversity is determined by ethnic background [21]. The null polymorphic variants (homozygous for nonfunctional allele)* GSTM1* and* GSTT1 *have been associated with a variety of health problems, such as high blood pressure in subjects from India and with the development of inflammatory bowel disease in non-Jewish patients from Israel [22, 23]. The allelic frequency of those genes showed considerable variation among Native Americans from Venezuela, where 15.2% of the Panare and 54.3% of Bari tribes presented* GSTM1* null genotype whereas none of the Pemon/Warao and 11.4% of Bari tribes presented* GSTT1 *null genotype [24].

In the Amazon, the allelic frequencies of the* GSTM1* and* GSTT1* null alleles have been identified in different Amerindians population [25]. The Mundurukus and Kayabi peoples are Amerindians that live along the Tapajós basin. There is little information about the social, cultural, and nutritional habits of these populations. However, it is well known that the Tapajós Basin has been historically impacted by gold mining activities since the late 1950s and that the native populations living around this area continue to be under risk of exposure to mercury intoxication. The social and health issues among the Mundurukus were evaluated by Nogueira [26]. This population is recognized for its innate ability to develop warfare and conquer new territories. As strategists, the Mundurukus occupied the major part of the Tapajós Valley (called Mundurukânia) and today they occupy only 04 villages (Nova Karapanatuba, Kato, Sai Cinza, and Missão São Francisco) comprising a total of 2,136 persons [26]. The association between molecular investigation and mercury concentration comprising a group of 117 Mundurukus showed that the monomorphic* GSTM1*+ allele is related to low levels (4.26 *μ*g/g) of mercury in hair samples. For comparison, 26% of subjects from the Kayabi community presented* GSTM1* null genotype and high levels (17.86 *μ*g/g) of mercury on hair, whereas the* GSTT1* null allele presented similar frequencies in both communities. Those achievements indicate that* GSTM1*+ allele would protect the individuals from mercury toxicity [27]. However, this study does not provide the period in which the blood samples were collected. This piece of information is important for studies in Mundurukus communities since fish are the main source of protein intake only during the dry season where the fishing practice is favorable in the Amazon [26]. The Kayabi community was also genotyped for other polymorphisms, such as manganese superoxide dismutase (*SOD2 Val-9Ala (T/C)*), catalase (*CAT 21A/T*), and glutathione peroxidase 1 (*GPX1 Pro198Leu (C/T)*) [28]. The results revealed a considerable variability of target gene frequencies among the Kayabi tribe, with these being 55% of* GSTM1* null, 45% of* GSTT1* null, 68% of* SOD2*, 26% of* CAT,* and 3% of* GPX1*. It was postulated that the Kayabi population, due to intense migration based on gold commerce, became somewhat mixed. However, assumptions based on the interethnic flux should be reviewed since migration does not always affect the genetic flow of Amerindians communities of Brazil. As an example, intercultural mixing was allowed by Mundurukus tribes, but matrimony between the members of the same family has historically been preserved [26]. Hence, connections between social, environmental, and genetic data are important for toxicological studies performed with the native population living in the Amazon since their behavior is also dictated by the environmental and economic conditions.

## 3. Immunotoxicology in Amazonian Populations

Autoimmune diseases (AID) comprise a class of clinical outcomes associated with an imbalance of the discrimination between self and nonself. The biological mechanisms associated with AID are poorly comprehended, but some general events can unleash the disease, such as pathogenic mechanisms, inflammation response, autophagy, and diet [29–31].

Genetic predisposition can contribute to the susceptibility to developing an AID. The hereditary studies and the discoveries of the molecular basis of AID have evolved and some researchers have already associated some genetic factors, such as the autoimmune polyendocrine syndrome type 1 (APS-1) and multiple sclerosis (MS), to disease development. The development of APS1, formerly autoimmune polyendocrinopathy-candidiasis-ectodermal dystrophy (APECED), is attributed to the loss of function mutation and dysregulation of the* AIRE *gene [32–34]. This gene codifies an autoimmune regulator protein (AIRE), which is a transcriptional factor that controls the expression of self-antigens in the thymus [35]. Different from APS1, multiple genes have been linked with the development of MS [36]. Among them, polymorphisms in the Human Leukocyte Antigen (HLA) class II genes have been extensively investigated [37–39]. Exposure to sunlight coupled with the intake of vitamin D has emerged as a factor to be involved with the development of MS and the advances in neuroepigenetic studies provide new clues about the plasticity and phenotype of the disease [40–43]. In spite of the progress of immunogenetics, no single gene has been identified as the main cause of the development of an AID. It is believed that AID is a conjunction of multiple genes working simultaneously to produce autoreactivity. Moreover, environmental triggers may play an important role in AID and, in conjunction with the genetic background, may determine the disease phenotype. However, identifying the criteria for an environmentally associated autoimmune disease is a challenge and the combination of complementary scientific areas may contribute to the improvement of such diagnosis [44]. The diagnosis of AID is complex and involves the conjunction of physical exam and a broad biochemical evaluation of the patient. The biochemical investigation usually includes common hematological routine studies (platelet and white blood cells count), coagulation tests, and urinalysis. Serum proteins, such as proinflammatory cytokines like IL-1, IL6, and TNF-*α*, can be useful since this can be correlated with any abnormal process caused by an AID, infection, or malignancies [45]. The presence of antinuclear autoantibodies (ANA) has been investigated as a biological marker for diagnosing an AID. The presence of ANA can be associated with a variety of AIDs, such as Hashimoto's thyroiditis; autoimmune hepatitis; and systemic lupus erythematosus [46–49]. However, health and alcoholic liver disease subjects also present positivity for a serologic test of ANA [50–52]. In this way, the lack of specificity of this marker has low clinical significance, but, in conjunction with other parameters, can establish such a diagnosis [53, 54].

A comparative analysis between three distinctive localities (Tabatinga, Jacareacanga, and Rio Rato, all of them located at Pará State) in Amazon revealed a high prevalence (51%) of detectable ANA at dilution 1 : 40 in workers from Rio Rato gold mining, who were exposed to mercury vapor (mean 4 *μ*g/L Hg in urine) [55]. At Tabatinga and Jacareacanga most of the population (>89%) had no detectable ANA (<1 : 10) in serum samples and only a small percentage (around 3%) had detectable ANA at ≥1 : 40 dilution. Those two communities were considered as a control since there was no prevalence of exposure to mercury vapor at the time of the study and they presented similar mercury concentrations in hair (6.4 *μ*g/g for Tabatinga and 8 *μ*g/g for Jacareacanga). However, 9.4% of the persons from Jacareacanga reported previous occupational activity as gold miners and 69.6% reported a history of past infection to malaria (among these 50% reported 2 or more infections). Moreover, a positive correlation between increased levels of ANA and malaria was related in persons from Jacareacanga that presented low concentration of mercury in hair (≤8 *μ*g/g). Highly prevalent malaria (90% of the subjects with malaria at the time of the study) was also reported for persons from Rio Rato whereas there was no prevalence of malaria at Tabatinga, but 10% of the persons reported previous infection. It is interesting to note that detection of ANA levels is accompanied by malaria infection (past or actual) even at localities that do not present any direct significant evidence of vapor mercury exposure (e.g., Jacareacanga). The correlation between the results of ANA and malaria cannot be considered insignificant and might open new insights into the contribution of malaria infections to the development of autoimmune dysfunction or the significance of ANA as biomarker to assess population based mercury exposure.

**Figure 1:**
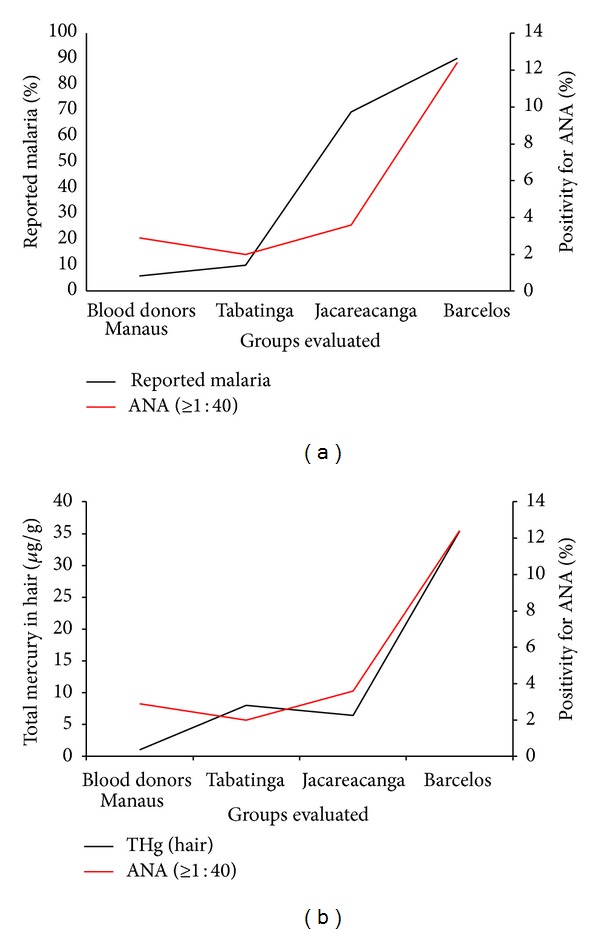
Correlation between malaria cases reported (a) and mercury levels in hair (b) with positive ANA.

The association between exposure to methylmercury from fish consumption and levels of ANA in serum was also investigated by Alves et al. [56]. The sample size was composed by 105 individuals from seven riverine communities that lived in Barcelos, a city in the Rio Negro basin, State of Amazonas. As the main occupational characteristic, they were classified as farmers and fish consumers (99% consume fish daily). Due to these characteristics, those individuals were identified as being exposed to methylmercury. At the time of the study 90.5% of the subjects had a history of malaria (but no prevalence). As inclusion criteria, all selected individuals had a minimum residence time equal to two years and had no clinical symptomatology of chronic diseases. As a control group, 105 blood donors from Manaus were selected, ranging from 18 to 50 years. In this group the patients were identified as low fish consumers with a poor history of malaria infection (5.7%). The riverine population presented higher levels of mercury (35.4 *μ*g/g) in hair than the control group (1.0 *μ*g/g). The results demonstrated a significant correlation between mercury levels in hair and fish consumption. The levels of ANA (≥1 : 40) were analyzed and the positivity among the riverine population was four times higher (12.4%) than in control the group (2.9%). The ANA positivity in Barcelos population has been cited by the authors as similar to Tabatinga population (2%) and Jacareacanga population (3.6%), where individuals were also characterized as higher fish consumers, according to Silva et al. [55]. However, we believe that this assumption may be contradictory, because the levels of ANA in Barcelos population were about four times higher than those of Tabatinga and Jacareacanga, showing a misleading comparison. Actually, ANA levels in the control group (blood donors) are more similar to those observed in Tabatinga and Jacareacanga populations. For comparison, Figure 1 exhibits the data obtained for all the population cited above according to (a) ANA associated with history of malaria and (b) ANA associated with mercury concentration in hair. From the compilation of data presented in Figure 1, the discussion referred to some important questions. (1) Tabatinga, Jacareacanga, and Barcelos populations are high fish consumers. However, the Barcelos population presented higher levels of mercury in hair than the others. Another distinguished feature of Barcelos is that almost all individuals reported past malaria infection. From this point of view, we could inquire: are the highest levels of ANA in Barcelos associated with increased levels of mercury or higher incidence of malaria? (2) The levels of ANA in Tabatinga and Jacareacanga population were similar to the control group (blood donors from Manaus). However, the control group presented the lowest level of mercury on hair. Thus, those results really reflect a correlation between mercury exposure and autoimmune dysfunction or are a reflection of a standard background value for ANA in the Amazonian communities? (3) The study performed at Rio Rato [55] was confronted with a population with high prevalence of malaria. This scenario differs from the other groups investigated (Tabatinga, Jacareacanga, Barcelos, and Blood Donors of Manaus) in which there was not a significant recorded prevalence of individuals with malaria at the time of research. In this way, is it possible to compare the results of ANA in a population with past exposure to malaria with a population that presented prevalent malaria at the time of the survey? In our opinion, it is mandatory to develop specific strategies in order to define the real contribution of malaria and mercury exposure to autoimmune dysfunctions.

Many aspects other than mercury exposure might contribute to the susceptibility to develop an AID at mining sites. Gardner et al. hypothesized that occupational and environmental conditions may contribute to autoimmune dysfunctions and that these could be determined by ANA/ANoA and mercury responsive cytokines (IFN-*γ*, IL-4, TNF-*α*, IL-1*β*, IL-1Ra, IL-10, and IL-17) in serum samples [57]. In order to understand if exposure to mercury vapor is a potential inductor of autoimmune dysfunction for human populations, three comparable mining activities (gold, diamond, and emerald) were studied and the similarities were determined according to social, environmental, and physical exertion factors. The areas of study were gold mining at Rio Rato (Pará), diamond mining at Davinópolis, and Santo Antônio Rio Verde (Goiás) and emerald mining at Itaobi and Vereador (Goiás). The highest mercury concentration (mean 3.67 *μ*g/L) was observed in urine samples of subjects from gold mining and a correlation was detected among those individuals that presented higher prevalence of detectable ANA and ANoA and higher concentration of IL-1*β*, TNF-*α*, and IFN-*γ*. No significant difference was observed between mercury concentration in hair samples of diamond (mean 0.72 *μ*g/L) and emerald (mean 0.279 *μ*g/L) mining workers. However, 33% of the workers from Davinopólis and 42% from Santo Antônio Rio Verde (both diamond mining sites) had malaria at the time of the study. Furthermore, 93% of the Rio Rato population also had prevalent malaria at the time of the study. The past exposure to vapor mercury was associated with dysfunction in the immune system, given that some subjects from diamond and emerald mines that presented detectable ANA or ANoA had reported previous gold mining activities. In spite of the importance of this data, this study does not consider that most of those workers also exhibit a past history of malaria too. In this context, it is well known that there is a high connection between gold mining activity and malaria infection in the Amazon [58–60]. In the Brazilian Amazon, it is important to note that malaria occurs frequently in adults, usually in males, whose professional activities, for example, gold mining, are associated with increased vulnerability to mosquitoes bites and the probabilities that those subjects have been infected by* Plasmodium *should be considered [58, 61]. In Brazil, the Amazon has the majority of malaria diagnoses and from 2000 to 2011* Plasmodium vivax* accounted for 78.7% of the registered cases [62]. The Amazon geographical characteristics (areas of forest, abundant rain, temperature, and humidity) propitiate proliferation of the malaria vector (*Anopheles*). Deforestation in the Amazon in order to create opening areas for gold mining activities is of the utmost importance [63]. This environmental problem causes disequilibrium in the biological cycle of the malaria vector that contributes significantly to proliferation by the mosquitoes [64]. The clinical symptoms of* P. vivax *infection differ and even asymptomatic subjects have been reported in the Amazon [65, 66]. However, fever, headache, and shivering are highly associated with disease manifestation [67–69]. Usually the clinical features are accompanied by elevated levels of TNF, IFN-*γ*, IL1, IL4, IL6, and IL10 in serum and/or plasma [70–73]. However, coinfection with other factors such as hepatitis B, polyparasitism, and additional tropical infectious diseases may be contributing to the clinical manifestation [74–78]. From the standpoint of Santos et al. and Marcano et al., the continuous activation of the immune system and possible overlapping of diseases may be the best profile to characterize manifestation of the disease in nonurban populations from the Amazon [58, 66]. Considering all the clues cited above, the association between mercury concentration and autoimmune dysfunction is unclear given that malaria infection occurs frequently among the Amazon gold mining workers and that it displays a broad of autoimmune biochemical parameters. Thus, it is important to understand the extent of immune imbalance that may be caused by malaria infection and to establish if mercury exposure acts as an additional factor to the development of autoimmune disease. Therefore, the evaluation of malaria infection associated with gold mining activities in Amazon is important prior to any suggestive association between mercury exposure and autoimmune dysfunction.

In order to study the association between mercury exposure and autoimmune diseases, Nyland et al. selected 61 pairs of women and their umbilical cords that were evaluated at three hospitals in Itaituba city (Pará State) in the years of 2000/2001 [79]. Variable sources of epidemiological parameters and sociodemographic variables, such as age, residence, occupation, family size, and social status, were collected. Additionally, reproductive and medical history, exposure to recent infections, smoking, alcoholism, drug use, diet, administration of medications during pregnancy, sex, and birth weight were also investigated. In spite of the epidemiological relevance of the data presented, the authors do not provide a discussion of all that information, excluding an important informative source (historical or disease prevalence) that would be helpful for contextualizing the discussion. The mercury was measured in maternal blood and umbilical cords as were other serum immunological markers, such as immunoglobulin (IgG1, IgG2, IgG3, IgG4, IgM, IgE, IgA, and IgG Total), cytokines (IL-1*β*, IL-1ra, IL-4, IL-6, IL-10, IL-17, IFN-*γ*, and TNF-*α*), and ANA and ANoA at 1 : 10 dilution. Statistical analysis showed a significant correlation between the levels of mercury in maternal blood and umbilical cord. Additionally, there was a correlation between certain types of immunoglobulins (total IgG total, IgG2, IgG3, and IgG4), cytokines (IL-1*β*, IL-6, and TNF-*α*), and levels of detectable ANA and ANoA. However, it was not possible to obtain a good correlation between mercury levels in the blood of mothers/umbilical cord with the target immunological biomarkers. The authors argue that the absence of a good correlation between mercury and immunological parameters may have been influenced by different histories of exposure of mothers and fetuses to mercury or even by the low size of the sample. Nevertheless, new perspectives for mercury research were suggested, such as increasing the size of the sample and the application of new proteomic technologies to aid in the identification of specific biomarkers. Despite discussions and appropriate suggestions for improvements that could help in the assessment for discovery of mercury biomarkers, the absence of specific correlations with other variables, such as tropical disease prevalence, may be masking the overlap profile of disease manifestation and, as consequence, immunological imbalance.

Nyland et al. selected 232 fish consumers with ages ranging from 15 to 87 years that were living in six communities in Itaituba city (historically known as a place where fish presented high levels of methylmercury due to gold mining activities in the Tapajós Valley) [80]. Mercury and selenium were measured in samples of blood, plasma, urine, and hair. Moreover, the positivity/negativity for ANA/ANoA at dilutions from 1 : 10 (1 : 10, 1 : 40, and 1 : 80) was also evaluated. The measurement of mercury allowed the creation of two groups of individuals: the first that presented high exposure to mercury (the fourth quartile) and the second that presented low exposure to mercury (the first quartile). Additionally, two other groups were created according to the immune response: the first that presented high positivity of detectable antibodies (ANA/ANoA+ to ≥1 : 80) and the second that presented negativity of detectable antibodies (ANA/ANoA- to <1 : 10). From the combination of these four groups only 96 individuals were analyzed for serum levels of interleukins (IL-1*β*, IL-1 receptor agonist, IL-1ra, IL-4, IL-6, IL-10, IL-17, IFN-*γ*, and TNF-*α*). The most relevant data were the identification of a positive and significant correlation between the levels of ANA positivity and high levels of mercury in blood, as well as the association between increased levels of pro- and anti-inflammatory cytokines (IL-4, IL-6, IL-17, and IFN-*γ*) with increasing levels of mercury on blood. However, the results showed decreased levels of pro- and anti-inflammatory cytokines (IL-4, IL-6, IFN-*γ*, and TNF-*α*) with increasing levels of mercury ANA-positive. The absence of other health information from the individuals made the results clinically limited, but the authors consider that methylmercury may act as a modulatory metal because it can broadly influence the development of an autoimmune response. Although the results of this study present the merit of contributing to the discussion of the use of biomarkers in response to exposure to environmental pollutants such as methylmercury, it seems to be too hasty to present them as a model, because there was not a more careful selection of the population exposed, no discussion of epidemiological and clinical aspects that could be influencing these results, and no comparison of the same immunological profile with an external control group (e.g., not exposed to methylmercury).

**Figure 2:**
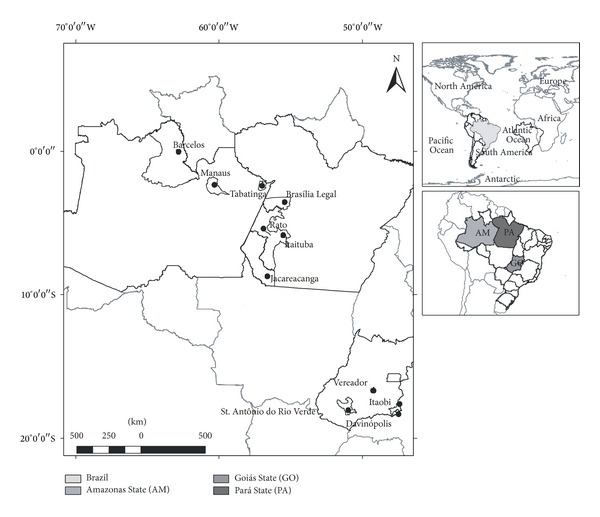
Map showing the locations where the studies about the association between mercury and immunological imbalance were performed.

The studies cited above covered eleven cities in the Brazilian Amazon (Figure 2) and gave a picture of the immunological status of some populations that presented different habits of fish intake, occupational activity, and environmental pressure. Thus, they highlighted the necessity for an application of a more comprehensive study about the immunological status of Amazonian population and opened new clues of the influence of malaria infection into immunological dysfunction.

## 4. Cytogenetic Studies

Chromatin is a dynamic structure which is under constant reorganization in order to attend to the cellular machinery [81]. Chromatin remodeling acts in tissue gene expression, as with IL-4, IL-13, and IFN-*γ* cytokine genes, in epigenetic regulation, and in the maintenance of nuclear DNA integrity [82–85]. Some intrinsic and extrinsic factors such as micronutrient availability, UV radiation, and ROS production can cause nuclear DNA damage [86–88]. Thus, DNA lesion must be accessible to the repair system (RS) in order maintain a constant genome viability [89]. The genomic lesions are of utmost importance since alteration in chromatin architecture, such as DNA double-strand breaks (DSBs), contributes to significant development of some diseases, such as cancer [90]. Thus, chromatin remodeling plays a central role in maintaining DNA integrity, given that it must allow the RS to function [91].

The cytogenetic damage caused by mercury has been reported since 1970 when Skerfving et al. reported a correlation between increased levels of mercury in blood of fish consumers and high frequency of chromosomal breaks [92]. On the other hand, the relation between occupational mercury exposure and cytogenetic damage is not so evident, as some exposed workers present normal chromosomal profile in lymphocytes [93].

In the Amazon, the methylmercury cytogenetic dysfunction was evaluated in blood sample of 250 adult fish consumers situated at Brasília Legal, a small locality in the Tapajós river basin [94]. The methylmercury exposure was measured by determining the inorganic mercury in hair samples. The median mercury concentration was 13.5 *μ*g/g, ranging from 0.57 to 71.85 *μ*g/g. In this study, three important parameters were evaluated: the mitotic index, the number of polyploidy aberrations, and chromosomal breaks. The study provides a clear correlation between high hair mercury concentration and decreasing lymphocyte proliferation (measured as mitotic index). Moreover, 86.7% of the studied population that presented mercury levels above 20 *μ*g/g had polyploidy aberrations and, among them, 37.9% presented chromatic breaks. It is important to note that 25.8% of the study participants reported past malaria infection and, among them, 79% had worked in gold mining region. However, the authors do not provide any information if malaria infection had contributed to the DNA damage. The results indicate that the impairment of lymphocyte proliferation could be useful as an initial biological sign of methylmercury poisoning. Following up the central idea, the authors raised the question of mercury exposure and its implications for immunology. Again, mercury, malaria, and immunological implications are somehow intertwined.

## 5. Future Perspectives

The lack of specificity in the determination of a biomarker for assessing mercury toxicity in humans follows the complexity of the metal's toxicology. This complexity is enhanced when Amazonian population is under investigation given that its diversity of health profiles, nutritional fluctuation according to seasonality, and cultural variety may lead to different mode of exposure. In this way, the studies of biomarkers in Amazonian population contributed significantly to the comprehension of the mercury exposure and added value for the comprehension of the health situation in which this population is submitted. However, in agreement with the multiplicity of this population, those studies provided confounding variables that should be studied in more detail in the future. As mentioned in the text, ANA is a screening test with low specificity, but, if positive, indicates the need for more extensive testing against other autoantigens (many of each do have disease specificity and severity association). This is one area that has not been explored in the Amazon but should as autoantibody profiling has the potential to discriminate between ANA of malaria versus mercury. Based on the assumption that the human metabolic route of inorganic mercury and methylmercury is far from being completely understood, basic questions concerning the mercury interaction in human body emerge as fundamental focus of research. Many studies at molecular levels have focused on the use of animal models or cell cultures for understanding mercury toxicity. However, mimetizing the human mercury toxicity and understanding the role of this compound speciation into cells and organisms are a challenge that may open new insights to our comprehension of the metabolic route of this compound. Methylmercury (the main known chemical form of exposure from diet) has high affinity for sulfhydryl groups. In the body, cysteine (present in protein structures) contains a thiol side chain which is formed by a carbon-bonded sulfhydryl group. Methylmercury has been found bound to some proteins (i.e., albumin and GSH). In this way, it becomes important to understand the biochemical interaction between proteins and mercury species with special attention to thiol-proteins. Finally, we believe that the high technology offered by the next generation sequencing and proteomics tools may open new insights into the biochemical route of mercury in populations with different ethnic background and exposure to different agents.
